# Main Risk Factors of Type 2 Diabetes Mellitus with Nonalcoholic Fatty Liver Disease and Hepatocellular Carcinoma

**DOI:** 10.1155/2021/7764817

**Published:** 2021-10-13

**Authors:** Yueying Qi, Lirong Fan, Decong Ran, Jieda Xu, Yuansong Wang, Jin Wu, Zhongyong Zhang

**Affiliations:** ^1^Department of Gastroenterology and Hepatology, Cangzhou Hospital of Integrated Traditional Chinese Medicine and Western Medicine, 31 West Huanghe Road, Cangzhou, Hebei, China; ^2^Department of Endocrinology, The Traditional Chinese Medicine Hospital of Botou, West Shengli Road, Botou, Hebei, China; ^3^Graduate School, Hebei University of Chinese Medicine, Xingyuan Road, Shijiazhuang, Hebei, China; ^4^Graduate School, Chengde Medical University, Anyuan Road, Chengde, Hebei, China; ^5^Department of Endocrinology, Hebei Province Cangzhou Hospital of Integrated Traditional Chinese Medicine and Western Medicine, 31 West Huanghe Road, Cangzhou, Hebei, China

## Abstract

Type 2 diabetes mellitus (T2DM) with nonalcoholic fatty liver disease (NAFLD) is a pathological metabolic disease characterized by high ketone lipid based on abnormal lipid metabolism. Compared with patients with single T2DM or NAFLD, T2DM complicated with NAFLD has more complicated pathogenic factors and pathological processes. Hepatocellular carcinoma (HCC), the leading malignancy arising from cirrhosis, is the second most lethal cancer globally. The purpose of this study was to clarify the main risk factors of T2DM with NAFLD and HCC. There are many challenges in the diagnosis and treatment of T2DM patients with NAFLD and HCC. The current gold standard is to adjust treatment strategy, optimize metabolic control, and improve liver phenotype. It is necessary to identify further the risk factors driving the progression of T2DM with NAFLD and HCC and evaluate new therapeutic targets, in addition to exploring the syndromic forms of T2DM combined with NAFLD and providing a theoretical basis for early prevention, diagnosis, and treatment of the disease using traditional Chinese medicine (TCM).

## 1. Introduction

Nonalcoholic fatty liver disease (NAFLD) is prevalent in patients with type 2 diabetes mellitus (T2DM) [1]. Previous studies had shown that 50% of T2DM patients had NAFLD, while the incidence of NAFLD in obese diabetic patients is as high as 100% [2]. There is increasing evidence that patients with T2DM have a particularly high risk of developing nonalcoholic fatty liver disease, nonalcoholic steatohepatitis, and hepatocellular carcinoma (HCC) [3]. HCC is a major life-limiting factor in progressive fibrotic liver disease, mainly caused by a chronic viral infection, alcohol abuse, and nonalcoholic fatty liver disease [4].

In prospective studies, preexisting diabetes mellitus was an independent risk factor for NAFLD progression and liver-related mortality [5, 6]. Studies had shown that the existence of NAFLD predicted the development of T2DM [7]. A cross-sectional study of T2DM patients found that the prevalence of NAFLD identified by ultrasound was 69% [8]. In a Swedish cohort study, most NAFLD patients (78%) were diagnosed with diabetes or impaired glucose tolerance at follow-up [9]. In addition, the interaction of environmental and genetic factors can promote the progress of T2DM with NAFLD. NAFLD increased the incidence of T2DM. At the same time, T2DM can effectively accelerate the development of NAFLD to a more serious form. In most developed countries, NAFLD is currently the most common liver disease and a major risk factor for HCC [10]. One study showed that diabetes increases the risk of HCC [11]. Whether the interaction between diabetes and the etiology of cirrhosis affects the risk of liver cancer remains controversial.

Although significant progress has been made in discovering new targets and treating chronic liver disease in recent decades, most treatment methods have not achieved satisfactory results [12]. Traditional Chinese medicine (TCM) treatment of the disease has the advantages of stable curative effect, safety, being nontoxic, low price, and multitarget effect [13]. In particular, the TCM syndrome types of different diseases may suggest different TCM treatment schemes.

## 2. Epidemiology of T2DM

The International Diabetes Federation estimates that 371 million adults worldwide had diabetes [14]. In China, the prevalence of diabetes reached 11.6% in 2010, affecting about 113.9 million adults [15]. It is estimated that, by 2040, about 642 million people will have diabetes, and T2DM is the main type of diabetes [16]. T2DM had become a heavy burden of limited medical resources. Since 1980, the incidence rate and prevalence of T2DM in the world had increased two times, and they are still increasing [17]. It had been reported that the prevalence of T2DM in women was on the rise globally, which was more common in low-income countries where obesity and aging were seen as driving forces [18]. In the United States, about one-third of patients with T2DM are adolescents [19]. It is estimated that the prevalence of T2DM in the population above 20 years of age ranges from 6.6% to 7.0% in Spain and 6.3% in Midi-Pyrénées, while the estimated value in men in these three regions is about more than 2% [20].

## 3. Epidemiology of NAFLD

NAFLD can be divided into two categories: primary and secondary. Fatty liver associated with metabolic syndrome caused by excess nutrition and cryptogenic fatty liver belongs to the category of primary nonalcoholic fatty liver disease. Fatty liver caused by malnutrition, total parenteral nutrition, drug/environment, and industrial toxicosis belongs to the category of secondary nonalcoholic fatty liver disease. NAFLD refers to all kinds of liver diseases, such as nonalcoholic steatohepatitis (NASH), simple steatosis (NAFL), and fibrosis. The incidence rate of NAFLD is expected to increase worldwide with the increase of obesity and diabetes. Recently, studies concluded that the prevalence of NAFLD worldwide is 25.2%, and the prevalence of NAFLD in the US is expected to increase by 50% by 2030 [21, 22]. The prevalence of NAFLD in China was about 20% [23]. NAFLD patients had not only a risk of progressive liver disease but also a significantly increased risk of cancer death [24]. NAFLD is diagnosed when more than 5% of liver cells show fat accumulation or by histological or imaging evaluation [25]. The pathogenesis of NAFLD is complex and has not been fully elucidated.

## 4. Epidemiology of HCC

Liver cancer mainly refers to malignant tumors originating from hepatocytes, liver epithelium, or liver mesenchymal tissue. HCC is more specific, mainly hepatocellular carcinoma. The etiology of the two cancers is also slightly different. Hepatocellular carcinoma is mainly caused by hepatitis B and hepatitis C. HCC accounts for >80% of primary liver cancers worldwide [26]. HCC accounted for 72.7% of global deaths in 2015 [27]. In addition, the World Health Organization (WHO) estimates that more than 1 million patients are expected to die from liver cancer within the next 10 years [28]. The incidence of liver cancer varies geographically, with the majority of liver cancer cases occurring in less developed regions, such as East Asia (54.8% of cases) and Southeast Asia (10.8% of cases) [29]. From 2006 to 2017, the incidence of HCC increased by 2-3% per year, mainly due to viral cirrhosis and a high incidence of NAFLD [30].

## 5. Main Risk Factors of T2DM with NAFLD

### 5.1. Genetic Factors

TM6SF2rs 58542926 mutation was closely related to NAFLD, age, body mass index (BMI), and T2DM [31]. TM6SF2 is located in ER and Golgi complex and has the function of mobilizing neutral lipids for VLDL assembly. In the absence of lipid droplets, lipids accumulate in the droplets [32]. However, the assessment of insulin resistance (IR) or oral glucose tolerance test did not reduce in the TM6SF2 gene mutation vector [33]. Therefore, the mutation may not be associated with IR.

Not only is PNPLA 3 gene mutation related to NAFLD, but also it has a slightly increased risk of T2DM [34, 35]. In fact, the expression of PNPLA 3 is directly regulated by the insulin regulatory transcription factor sterol regulatory element-binding protein 1c (SREBP-1c). In the case of obesity and IR, the accumulation of pathogenic PNPLA 3 mutation products aggravates liver steatosis, inflammation, and cirrhosis [36].

Adiponectin (HMW) is an adipocytokine and insulin-sensitive substance, which plays an essential role in the pathogenesis of diabetes mellitus and NAFLD [37]. HMWrs 266729 polymorphism is associated with an increased risk of NAFLD patients [38]. Studies on different populations showed that HMW gene polymorphism affected the development of NAFLD [39, 40]. There was a significant correlation between rs1501299 and NAFLD in some female diabetic patients in Japan [41]. HMW is considered a potential biomarker for the detection and prediction of NAFLD complicated with T2DM [42]. Lu et al. found that the mutation frequency of LEPR nucleotide 3057 G > A (rs1805096) was 76.0% in 104 T2DM patients with NAFLD. The results suggested that LEPR gene G3057 A (rs1805096) polymorphism may be involved in NAFLD by regulating lipid metabolism and affecting insulin sensitivity in patients with T2DM [43].

### 5.2. Insulin Resistance

The close relationship between NAFLD and T2DM is that they have common pathogenesis, namely, IR [44]. IR refers to the decrease of tissue response to insulin [45]. The pathogenesis of NAFLD is described as the “multiple hit hypothesis.” IR plays a central role in the first attack, resulting in an imbalance between factors that promote liver fat accumulation and factors that prevent fatty acid accumulation [46, 47]. The steady-state model assessment value of *β* cell function and the decreased value of *β* cell function in patients of T2DM with NAFLD were higher than those in patients without NAFLD, including IR of liver and adipocytes [48]. Therefore, NAFLD often coexists with T2DM.

Swollen and inflamed visceral adipose tissue is likely to trigger various factors that may be correlated with the development of IR and NAFLD, such as inflammatory adipocytokines and free fatty acids [49]. The interaction between hepatic steatosis and IR establishes a circle to promote the development of T2DM and NAFLD. In addition, glucose cotransporter 2 can promote renal reabsorption of glucose and reduce urinary glucose excretion by increasing blood glucose and body weight (BW), thus aggravating IR in T2DM and NAFLD patients [50]. This relationship between T2DM, IR, and NAFLD is believed to be due to insulin being delivered directly to the portal vein after secretion in the same way as glucose absorbed. IR plays a crucial role in the pathogenesis of T2DM with NAFLD. Therefore, insulin sensitizer is considered as an effective treatment.

### 5.3. Lifestyle

A multicenter clinical trial involving 5145 overweight adults with T2DM showed that, after 12 months of intensive lifestyle intervention, steatosis and NAFLD were significantly reduced, and weight loss was at least 7% [51]. Recent randomized controlled trials (*n* = 154) had again shown that lifestyle intervention could effectively alleviate NAFLD in nonobese and obese patients [52]. Numerous studies have shown that developing a reasonable exercise plan is significant for alleviating T2DM and NAFLD. Reasonable exercise plays an important role in controlling the blood sugar and blood lipids of patients and can significantly improve the therapeutic effect of T2DM or NAFLD.

The relationship between diet and T2DM with NAFLD is very complex. Excess of total energy intake can lead to obesity by changing the energy balance. A high carbohydrate diet (50% to 65% of carbohydrate calories) is associated with IR and obesity [53]. All of these are risk factors for damaging NAFLD phenotype and increasing IR [25]. A review study evaluated the effects of probiotics and synbiotics on obesity, T2DM, and NAFLD [54]. The beneficial effects of probiotics and synbiotics improved liver function and metabolic parameters in NAFLD patients.

Lower frequency and level of physical activity and being sedentary for a long time were associated with IR, T2DM, and NAFLD. Sedentary behavior is associated with chronic low-grade inflammation and can lead to obesity [55]. Exercise management can prevent or delay the progress of T2DM [56]. Among large numbers of middle-aged Korean people, being sedentary and reduced physical activity are positively correlated with the prevalence of NAFLD, which supports the importance of increasing physical activity to promoting physical activity [57, 58].

### 5.4. Obesity

Obesity is a chronic metabolic disease, which is mainly characterized by excessive accumulation of fat and overweight. The current research showed that the causes of obesity are diverse, and the main reasons are divided into congenital factors and exogenous factors. Studies have shown that the congenital factors of obesity are mainly genetic factors, while the exogenous factors are mainly excessive diet, lack of exercise, or pathological obesity.

The incidence rate of obesity and its metabolic complications worldwide had risen sharply in recent years. Obesity is an important risk factor for NAFLD and T2DM and may provide a common link through IR [59]. Recent studies had shown that obesity (whether peripheral or central obesity) usually preceded NAFLD, and NAFLD preceded the development of T2DM [60]. Obesity is closely related to adipose tissue dysfunction in NAFLD patients, which may accelerate IR and pancreatic *β* cell dysfunction [61]. To a large extent, IR in obese patients is the result of adipose tissue inflammation and adipocyte regulation disorder [62]. Weight loss has a significant effect on T2DM with NAFLD, and the weight loss is mainly due to the reduction of fat mass, especially visceral fat, rather than skeletal muscle mass [63]. Bariatric surgery is an effective method to treat obesity, which has been proved to significantly improve or even cure diabetes and improve the histological characteristics of NAFLD [64].

In addition, a large number of studies have shown that obesity is closely related to the intestinal flora. The change of intestinal microbiota composition has been considered an effective therapy to regulate obesity [65].

### 5.5. Others

The data showed that NAFLD and diabetes were related to the decrease of CYP3A4 activity in the liver [66]. In human studies, low plasma adiponectin levels are associated with an increased risk of T2DM, and low adiponectin levels are an independent risk factor for NAFLD [67]. In addition, LDL-c, FPG, BMI, FINS, TC, and HOMA-IR were also risk factors of T2DM with NAFLD [44]. In 146 T2DM patients with NAFLD, multivariate analysis showed that dyslipidemia, elevated LDL, HbA1c, and diastolic blood pressure were risk factors [68]. In addition, human and animal intestines are occupied by a variety of microorganisms. These microorganisms play a key role in maintaining intestinal function and regulating host immune response and chronic diseases such as obesity, diabetes, and NAFLD [69–71].

## 6. Main Risk Factors of HCC

Major risk factors contributing to the rise in HCC include high prevalence of HBV and HCV infection, followed by an increased incidence of alcohol abuse, obesity, NAFLD, and uncontrolled type 2 diabetes [10]. In areas with high incidence, 80% of HCC patients test positive for hepatitis B surface antigen (HBsAg) in serum [72]. Moreover, 10–20% of patients with hepatitis B can develop HCC without cirrhosis [73]. Hepatitis C virus (HCV) infection is also a major risk factor for HCC, which leads to a 5- to 20-fold risk of HCC [74]. Indeed, persistent cellular stress, repeated necrosis, and compensatory regeneration of cells, as well as chronic inflammation, lead to cellular senescence and mutagenesis, ultimately leading to hepatocarcinogenesis [75]. The mechanism of NAFLD-induced HCC is not fully understood, and there is no way to prevent NAFLD patients from progressing to HCC [76]. T2DM is a risk factor for NAFLD and increases HCC incidence two- to threefold [77]. NAFLD HCC patients have increased levels of IL-13, which can activate myeloid-derived suppressor cells and promote tumor progression by suppressing tumor immunity [78]. Another mechanism underlying NAFLD HCC is PNPLA3 gene polymorphism, possibly related to by enhancing inflammatory signaling [79].  Table 1 compiles the principal observational investigations and meta-analyses analyzing the association between T2DM and the risk of HCC.

## 7. TCM Syndrome Types of T2DM with NAFLD

In recent years, TCM and its extracts have been considered a new potential source of therapeutic drugs for preventing and treating fatty liver disease [80]. According to modern TCM theory, type 2 diabetes belongs to the category of diabetes. There are many problems, such as dryness and heat injury, qi and yin deficiency, liver qi and yin deficiency, liver failure, spleen failure, liver blood deficiency, and spleen stomach heat accumulation. Therefore, it can be divided into eight types: stomach heat syndrome, lung dryness syndrome, spleen qi deficiency syndrome, lung qi deficiency syndrome, yin and yang deficiency syndrome, kidney yin deficiency syndrome, blood stasis syndrome, and phlegm retention syndrome.

Statistical analysis showed that spleen deficiency syndrome was the main syndrome type in T2DM with the NAFLD group [81]. This has also been confirmed in other studies, and phlegm is one of the main syndrome characteristics [82]. There are also studies suggesting that, in T2DM patients with NAFLD, the proportion of damp-heat accumulation is the highest, followed by yin deficiency heat [83]. Additionally, studies also found that damp-heat trapped spleen syndrome and qi and yin deficiency syndrome are the most important syndrome types [84]. Correct evaluation of TCM syndrome types is helpful to improve the clinical effect of TCM combined with general therapy in the treatment of T2DM with NAFLD.

## 8. Conclusion and Future Prospect

**Figure 1:**
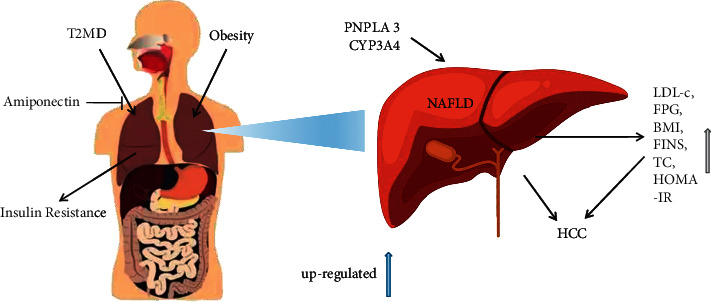
Biological mechanisms linking type 2 diabetes mellitus and NAFLD.

At present, T2DM with NAFLD is considered as a multifactorial disease with genetic and environmental factors. IR is considered as a key risk factor for the occurrence and development of T2DM with NAFLD. IR in the peripheral tissue and liver is one of the causes of this condition, leading to the increase of circulating glucose and lipid substrates for lipid accumulation in the liver. Changing diet structure is beneficial to delay the progression of T2DM with NAFLD. It supports more extensive application of traditional Chinese medicine, Chinese patent medicine, and acupuncture physiotherapy, which provides theoretical support for the clinical application of traditional Chinese medicine therapy. However, there are still many deficiencies in the treatment of TCM. Therefore, further research and clinical verification are needed (Figure 1).

Prevention and treatment of viral hepatitis and NAFLD were vital factors in reducing the global burden of liver cancer. Implementation of screening for viral hepatitis and surveillance for hepatocellular carcinoma in high-risk patients are essential to improve current poor outcomes for patients with HCC. However, a better understanding of risk factors for liver cancer is required for developing new effective regimens and improving the efficacy of the existing therapies. [85]

## Figures and Tables

**Table 1:** Studies which have evaluated the association between type 2 diabetes and risk of HCC.

Study	Study characteristics	Diabetes diagnosis	Covariate adjustment considered	Main findings
Huo et al., Eur J Gastroenterol Hepatol 2003; 15 : 1203-8	Prospective study: 239 HCC patients (16.3% of whom had DM). Mean follow-up: 2.6 years	Fasting glucose ≥126 mg/dL or 2-hour postload glucose ≥200 mg/dL, or past history	Age, sex, tumor size, anti-HCV-Ab positivity, HBeAg-positivity, cirrhosis, alcohol intake, alpha-fetoprotein, albumin, bilirubin	DM did not affect long-term survival in HCV-related HCC but was a recurrence-independent prognostic factor for HBV-related HCC
Coughlin et al., Am J Epidemiol 2004; 159 : 1160-7	Population cohort study: 467,922 men and 588,321 women without history of cancer at baseline. Mean follow-up: 16 years	Self-reported	BMI	DM was associated with increased risk of incident HCC only in men
El-Serag et al., Gastroenterology 2004; 126 : 460-8	Prospective study: 73,643 patients with DM and 650,620 patients without DM. Mean follow-up: 5 years	Self-reported	Alcoholic liver disease, viral chronic hepatitis, demographic variables	DM was associated with an increased risk of incident HCC. DM carried the highest risk among patients with a follow-up longer than 10 years
Davilla et al., Gut 2005; 54 : 533-9	Population-based case-control study: 2,061 HCC patients (of whom 43% with DM) and 6,183 noncancer controls (of whom 19% with DM)	Electronic register	Age, sex, race, HCV, HBV, alcoholic liver disease, and hemochromatosis	DM was associated with a nearly threefold increased risk of HCC
Inoue et al., Arch Intern Med 2006; 166 : 1871-7	Prospective study: 97,771 Japanese adult individuals followed up for cancer incidence over 5 years. At baseline, 4.7% of them had DM	Self-reported	Age, study area, BMI, prior cardiovascular disease, smoking, alcohol intake, leisure-time physical activity, green vegetable intake, coffee intake	DM was associated with increased risk of total cancer and cancer in specific sites, including HCC
El-Serag et al. Clin Gastroenterol Hepatol 2006; 4 : 369-80	Meta-analysis: a total of 26 studies (of which 13 were case-control studies and 13 were cohort studies), inclusive of approximately 3 million individuals	Self-reported	Alcohol intake, chronic viral hepatitis, diet, BMI	Among 13 cohort studies, DM was associated with an increased risk of HCC
Kawamura et al., J Gastroenterol Hepatol 2008; 23 : 1739-46	Prospective study: 40 consecutive HCC patients (with HCC associated with non-B, non-C hepatitis) and later underwent surgical resection or radiofrequency ablation. Prevalence of DM was 45%. Mean follow-up: 5 years	Fasting glucose ≥126 mg/dL or past history	Age, sex, dyslipidemia, smoking, alcohol intake, history of blood transfusion, state of liver disease (chronic hepatitis or cirrhosis), AST, albumin, bilirubin, alpha-fetoprotein, prothrombin time, tumor size, multiplicity, hypervascularity, and portal vein invasion of HCC	DM was a significant predictor of tumor recurrence after potentially curative therapy for HCC
Donadon et al., World J Gastroenterol 2009; 15 : 2506-11	Case-control study: 465 HCC patients, 618 with cirrhosis, and 490 control subjects. The prevalence of DM was 31.2% in HCC, 23.3% in cirrhotic patients, and 12.7% in control group	Self-reported	Age, sex, BMI, alcohol abuse, HBV, and HCV	DM was an independent risk factor for HCC. Among male patients with DM, there was a positive association of HCC with insulin/sulphonylurea treatment and an inverse association with metformin
Hassan et al., Cancer 2010; 116 : 1938-46	Hospital-based case-control study: 420 patients with HCC and 1,104 healthy controls. The prevalence of DM was 33.3% in patients with HCC and 10.4% in controls	Self-reported	Age, sex, race, educational level, smoking, alcohol intake, HCV, HBV, family history of cancer	DM increased the risk of HCC. Treatments with sulfonylureas or insulin were associated with higher HCC risk, whereas treatments with metformin or glitazones were associated with lower HCC risk
Hense et al., Diabetol Metab Syndr 2011; 3 : 15	Community-based study: 26,742 DM patients, who were 40 to 79 years old and resided in the Muenster district. Mean follow-up: 3.3 years	Self-reported	Sex, diabetes duration, BMI, insulin treatment	Risk of any incident cancer in DM was increased, in particular for HCC. Insulin therapy was related to higher cancer risk, while metformin was not
Johnson et al., Diabetologia 2011; 54 : 2263-71	Population-based retrospective cohort study: 185,100 individuals with DM and 185,100 without DM, matched by sex and age. Mean follow-up: 10 years	Electronic register	Age, sex, socioeconomic status, number of physician visits, year of diagnosis	DM was associated with increased risk of selected cancers, including HCC
Li et al., Int J Canc 2012; 131 : 1197-202	Hospital-based case-control study: 1,105 patients with HBV-related HCC and 5,170 patients with chronic HBV. The whole prevalence of DM was 6.7%	Fasting glucose ≥126 mg/dL or past history	Age, family history of HCC, city of residence, HBV-Ag, and cirrhosis	DM was associated with increased risk of HCC, only in women
Wang et al., Int J Cancer 2012; 130 : 1639-48	Meta-analysis: a total of 25 cohort studies, enrolling 1,283,112 persons. Mean follow-up: 8.8 years	Self-report, medical records	Geographic location, alcohol intake, history of cirrhosis, or HBV and HCV infections	DM was associated with increased risk of incident HCC and higher HCC mortality. Longer diabetes duration and use of sulfonylureas or insulin were associated with increased risk of HCC. Metformin treatment was protective
Wang et al., Diabetes Metab Res Rev 2012; 28 : 109-22	Meta-analysis: 17 case-control studies (a total of nearly 6,000 HCC cases and 74,000 controls) and 32 cohort studies (a total of nearly 6,500,000 individuals)	Self-report, medical records	BMI, prior hepatitis, cirrhosis, alcohol intake, smoking, treatment, duration of diabetes	The combined risk estimate of all studies showed a significant increased risk of HCC among DM individuals. In addition, meta-analysis of 7 cohort studies found a significant increased risk of HCC mortality for individuals with DM compared to those without
Lai et al., Am J Gastroenterol 2012; 107 : 46-52	Population-based cohort study: 19,349 newly diagnosed DM patients and 77,396 control subjects without DM. Mean follow-up: 5 years	Electronic register	Age, sex, cirrhosis, alcoholic liver damage, viral hepatitis	DM was associated with increased risk of incident HCC. Use of metformin or glitazones was associated with reduced HCC risk
Schlesinger et al., Ann Oncol 2013; 24 : 2449-55	Community-based cohort study: 363,426 participants, after excluding those with cancer at baseline. Mean follow-up: 8.5 years	Self-reported	Age, sex, center, education level, smoking, alcohol intake, BMI, waist-to-height ratio	DM was independently associated with higher risk of incident HCC and biliary tract cancer. HCC risk was higher in those treated with insulin. Results were similar in HCV/HBV-negative individuals
Zheng et al., PLoS One 2013; 8:e84776	Hospital-based retrospective case-control study: 1,568 participants of whom 716 patients were diagnosed with benign liver diseases and 852 patients were diagnosed with HCC. The prevalence of DM was 7.6%	Fasting glucose ≥126 mg/dL or 2-hour postload glucose ≥200 mg/dL, HbA1c ≥ 6.5%	Age, sex, HBV and HCV infections, cirrhosis, gallstone disease, cholinesterase, alkaline phosphatase	DM was associated with increased risk of HCC. However, there was a significant interaction between DM and HBV on HCC occurrence
Koh et al., Br J Cancer 2013; 108 : 1182-8	Community-based cohort study: 63,257 middle-aged and older individuals. The prevalence of DM was 8.6%. Mean follow-up: 14 years	Self-reported	Age, sex, BMI, recruitment year, education level, smoking, alcohol intake, consumption of coffee and tea	DM was associated with an increased risk of incident nonviral HCC
Miele et al., Gastroenterol Res Pract 2015; 2015 : 570356	Hospital-based case-control study: 224 HCC patients and 389 controls. The prevalence of DM was 19.7%	Self-reported	Age, sex, smoking, alcohol intake	DM was associated with increased risk of HCC. Treatment with any glucose-lowering drugs was not associated with increased HCC risk
Adami et al., J Natl Cancer Inst 1996; 88 : 1472-7	Hospital-based cohort: 153,852 patients with DM. Follow-up: from 1 to 24 years	Hospital discharge diagnosis	None	DM was associated with increased risk of incident HCC
La Vecchia et al., Int J Cancer 1997; 73 : 204-7	Case-control study: 428 HCC cases, 59 with gallbladder and bile duct cancers, and 1,502 control subjects from hospital	Self-reported	Age, sex, area of residence, education level, alcohol intake, BMI, smoking, history of chronic hepatitis and cirrhosis, family history of liver cancer	DM was associated with increased risk of incident HCC
